# Identifying and handling unbalanced baseline characteristics in a non-randomized, controlled, multicenter social care nurse intervention study for patients in advanced stages of cancer

**DOI:** 10.1186/s12885-022-09646-6

**Published:** 2022-05-18

**Authors:** Johann Frick, Pimrapat Gebert, Ulrike Grittner, Anne Letsch, Daniel Schindel, Liane Schenk

**Affiliations:** 1 Charité – Universitätsmedizin Berlin, corporate member of Freie Universität Berlin, Humboldt-Universität zu Berlin, and Berlin Institute of Health, Institute of Medical Sociology and Rehabilitation Science, Charitéplatz 1, 10117 Berlin, Germany; 2 Charité – Universitätsmedizin Berlin, corporate member of Freie Universität Berlin, Humboldt-Universität zu Berlin, and Berlin Institute of Health, Institute of Biometry and Clinical Epidemiology, Charitéplatz 1, 10117 Berlin, Germany; 3 Berlin Institute of Health (BIH), Anna-Louisa-Karsch-Str. 2, 10178 Berlin, Germany; 4 Department of Medicine II, Hematology and Oncology, University Hospital Schleswig-Holstein, Arnold-Heller-Straße 3, 24105 Kiel, Germany; 5 Charité – Universitätsmedizin Berlin, corporate member of Freie Universität Berlin, Humboldt-Universität zu Berlin, and Berlin Institute of Health, Charité Comprehensive Cancer Center, Charitéplatz 1, 10117 Berlin, Germany

**Keywords:** Advanced cancer, Nurse support, Quality of life, Patient-reported outcomes, Intervention study, Recruitment phase, Selection effects, Study participation

## Abstract

**Purpose:**

Given the psychosocial burdens patients in advanced stages of cancer face, innovative care concepts are needed. At the same time, such vulnerable patient groups are difficult to reach for participation in intervention studies and randomized patient inclusion may not be feasible. This article aims to identify systematic biases respectively selection effects occurring during the recruitment phase and to discuss their potential causes based on a non-randomized, multicenter intervention study with patients in advanced stages of cancer.

**Methods:**

Patients diagnosed with at least one of 16 predefined cancers were recruited at four hospitals in three German cities. The effect of social care nurses’ continuous involvement in acute oncology wards was measured by health-related quality of life (EORTC QLQ-C30), information and participation preferences, decisional conflicts, doctor-patient communication, health literacy and symptom perception. Absolute standardized mean difference was calculated as a standardized effect size to test baseline characteristics balance between the intervention and control groups.

**Results:**

The study enrolled 362 patients, 150 in the intervention and 212 in the control group. Except for gender, both groups differed in relevant socio-demographic characteristics, e.g. regarding age and educational background. With respect to the distribution of diagnoses, the intervention group showed a higher symptom burden than the control group. Moreover, the control group reported better quality of life at baseline compared to the intervention group (52.6 points (SD 21.7); 47.8 points (SD 22.0), ASMD = 0.218, *p* = 0.044).

**Conclusion:**

Overall, the intervention group showed more social and health vulnerability than the control group. Among other factors, the wide range of diagnoses included and structural variation between the recruiting clinics increased the risk for bias. We recommend a close, continuous monitoring of relevant social and health-related characteristics during the recruitment phase as well as the use of appropriate statistical analysis strategies for adjustment, such as propensity score methods.

**Trial registration:** German Clinical Trials Register (DRKS-ID: DRKS00013640); registered on 29th December 2017.

**Supplementary Information:**

The online version contains supplementary material available at 10.1186/s12885-022-09646-6.

## Background

Cancer diagnoses burden patients with extensive medical procedures and have a far-reaching impact on all individual life contexts [1]. Oncological diseases are prevalently associated with psychological stress, and related mood disorders such as depression, but also anxiety disorders and insomnia are known to occur as complications in cancer patients [2, 3]. Depending on the type of cancer and the stage of the disease, there is a high incidence of disability, which contributes to a low quality of life [4]. Medical care of psychosocial burdens of disease in the context of a cancer diagnosis are heterogeneously dealt with and distinct services are provided. The spectrum ranges from state-funded health services and mental health case managers to specific offers aimed at more effective patient-provider communication to achieve improved care outcomes [5, 6].

### Difficulties in accessing appropriate end-of-life care

Studies evaluating palliative care interventions in patients with advanced cancer could demonstrate positive effects of early palliative care. Thereby, compared to patients provided with standard care, patients receiving early palliative care services showed an increased overall quality of life (QoL), a better perception of the own care situation as well as a longer life expectancy, while they were less prevalently diagnosed with depression and anxiety disorders [7, 8]. Moreover, family members experienced the process of dying as less painful if their deceased patients had received more than 22 days of palliative or hospice care as compared to relatives of patients with shorter care intervals [9].

Nevertheless, not all patients are provided with such kind of end-of-life care, as a US American study emphasizes, in which only a small proportion of patients had received palliative or hospice care [10]. Hence, several barriers to access appear to exist. A systematic review identified patients’ sociodemographic and socioeconomic characteristics (e.g. gender, origin, and housing situation) as well as communication problems with the care institutions to be essential factors that minimize access to palliative and hospice care [11]. Furthermore, existing barriers to care access may be explained by the often prevailing curative-oriented treatment approaches patients are faced with until the very end of their lives [12]. At the same time, patients’ ability to assess the severity of their disease has shown to be limited. For instance, a study on patients with colorectal or lung cancer showed that about two-thirds of all patients were not aware that the likelihood of cure by means of chemotherapy is relatively low [13]. Moreover, limited care capacity further limits access to palliative care or inpatient hospices [14].

### Additional types of care to overcome sector boundaries in the healthcare system

Given the psychosocial burdens, various deficiencies in health care systems, and the QoL benefits of empowering patients in advanced cancer stages, there is a need of additional care concepts [15]. For example, patients and their families appear to require specific information for medical care and for post-discharge care during inpatient oncology treatment [16]. Although such types of support services do exist within the German care context (e.g. social services), those are currently only available at the time of hospitalization. Standardized follow-up care in the outpatient setting is lacking [4]. Supportive care services have become increasingly important for patients to be able to engage in and manage their care more independently [17]. Characteristic to such programs is specialized trained staff, who are in regular contact with patients. Thereby, the personnel aims to decrease psychological distress and improve QoL by navigating their patients through the fragmented health care systems [18, 19]. The effectiveness of previous navigation interventions has been controversially discussed with respect to the stage of disease and the specific diagnoses [19–22].

### Combination of psychosocial care and navigation

The intervention to be tested in the OSCAR study (acronym for Oncological Social CARe project) combines the use of well-trained nurse navigators with a strong focus on coordinating psychosocial counselling of patients. Previous supportive navigation approaches that start at earlier stages of the disease [19] or are only available in the context of inpatient treatment [17] or have a quite limited duration of care [17, 19] or are based on nonmedical personnel [22, 23] are further developed. Based on a curriculum of the Saxon Cancer Society a specific care concept for patients in advanced stages of cancer was designed. The concept includes a close-meshed care and nurse-based approach. For a period of 12 months patients and their relatives receive monthly regular counseling sessions during which they are supported with respect to psychosocial, medical as well as social security related issues to facilitate the path through the health system [24]. To this end, social care nurses (SCN) visit or call patients regularly and assess various dimensions of their QoL using a structured questionnaire. While OSCAR aimed to overcome existing gaps in the German cancer care and appears promising in improving patients’ QoL, research on the effectiveness of this care program is needed—as with other innovative approaches.

### Challenges in intervention studies with vulnerable populations

Evidence of effectiveness is a prerequisite for a broad implementation of innovations in health care systems, but participation rates are low overall and studies show selection effects [25]. Studies that examined participation behavior in intervention trials based on responder-non-responder comparisons identified tumor stage, lymph node involvement and comorbidities as influencing participation [26, 27]. In addition, convenience, the expected success of treatment and side effects were identified as important factors for acceptance and participation in a recommended therapy and clinical trials [28, 29]. The treatment experiences of significant others were also influential in the participation decision [28]. This points to challenges inherent in trials in vulnerable patient populations as those with advanced cancer and poor prognosis. In addition to the described selection effects on the patient side, restrictive funding conditions, particular research questions, time restrictions or medical treatment reality can impede randomization in the recruitment and thus increase the risk of selection bias. Regardless of whether randomization is used or not, control of baseline distribution of covariates appears to be recommended [30, 31]. Further, when performing the analyses prespecified baseline covariates should be included to ensure that imbalances between intervention and control groups based on chance do not affect effect estimates [31, 32]. Non-randomized studies are inherently more susceptible to bias, due to a higher risk of systematically differences between intervention and control groups. To investigate and address recruitment based selection bias different approaches have been discussed before [33–35].

This article aims to identify systematic biases respectively selection effects occurring during the recruitment phase in a non-randomized intervention study with patients in advanced stages of cancer by performing comparative analyses of the baseline data. In addition, we will shed light on mechanisms and potential causes for bias and discuss suitable compensation strategies in order to improve future analyses and recruitment practices.

## Methods

### Study design

The OSCAR-study was designed as a non-randomized, controlled, multicenter trial to assess the effect of the social care nurse intervention. Data were collected from February 2018 to February 2020 [24]. Four clinics in three German cities served as recruitment channels, of which two belonged to university hospitals. The other two were maximum and standard care hospitals, that also provide a comprehensive and differentiated range of services as well as appropriate medical and technical facilities fulfilling supra-local priority tasks. The health insurers participating in the project show regionally very different shares of insured patients. By means of a Germany-wide patient potential analysis based on the defined cancer entities, the hospitals with the greatest patient potential were identified and recruited for the study.

### Study population

Inclusion criteria for patients were defined as follows: ≥ 18 years of age, a combination of at least one of 16 types of cancer in combination with a burdensome therapy (e.g., surgical operation, radiotherapy, cytotoxic chemotherapy, etc.) (see Additional file 1: ICD-10 codes and OPS codes). Moreover, membership in one of 37 predefined German statutory health insurance companies (out of a total of 102 statutory health insurance funds in Germany) was a prerequisite for participation in the intervention group. All of the 37 health insurance funds have a common historical background, since as company health insurance funds they exclusively insured the employees of a particular industrial company or group. Patients who were not member with one of the predefined insurance companies were recruited into the control group. Exclusion criteria were advanced dementia and acute addiction. After providing written consent, patients were interviewed by the study team using a paper questionnaire for the scientific evaluation.

### Study intervention

The intervention was provided as an additional service alongside the patients’ regular oncological care [24]. Following Kelly et la., the activities and roles of the social care nurse can be described as follows [36]. The nurses were employed by the four participating hospitals and worked in regular shifts on the oncological wards. In addition, they were given a fixed quota of hours to function as social care nurses.

At least once a month patients were actively contacted by their personal social care nurse. The contact was via the telephone, email in or a face-to-face meeting in the hospital. The meetings were to assess patient needs and identify gaps in care by using the cancer-specific quality of life questionnaire (EORTC QLQ-C30) [37]). Key functions of the social care nurses were to screen for support needs and to provide assistance in coordinating medical, psychosocial and palliative support services. Additional functions were to educate patients about the healthcare system (e.g., application for assistance) and navigate services (e.g., contact to therapists, support groups, early palliative care) to reduce barriers to receiving timely services. All social care nurses had at least five years of professional experience as trained nurse. The majority had an additional training in psycho-oncology. In preparation for their role, the SCNs received further three weeks of full-time training including: e.g., knowledge about tumor diseases, therapy options as well as special issues of oncological care and palliative medical services, knowledge of psychological aspects of the diseases (side-effect management, pain therapy, nutritional and wound therapy), psycho-oncology incl. dealing with grief and processing strategies as well as the inclusion of intercultural peculiarities, information on social security support and care services for affected persons and their relatives, theory and practice of participatory decision-making.

### Patient-reported outcome measures

The primary outcome – quality of life (QoL) – was assessed by the EORTC QLQ-C30. The average of the global health status scale and the quality of life scale was used as the key scale. Scores ranges between 0 and 100. Higher scores indicate a better QoL [37, 38]. For secondary outcomes, patients’ health literacy was assessed by means of a validated assessment tool (HLS-EU-Q6 [39], score ranges between 1 and 4; score is grouped into insufficient (1–2 score), problematic (2–3 score), and sufficient (3-4 score). The relationship between physicians and patients was measured by the quality of the doctor-patient communication using an adapted version of the PRA-D (score ranges from 5 to 35; higher scores indicate better doctor-patient communication) [40]. Information and participation preferences (API-DM) were surveyed using the modified German version of the Autonomy Preference Index [41] (score ranges from 0 to 100; higher scores indicate a greater preference of information or participation). Moreover, the Decisional Conflict Scale (DCS, [42]) was utilized for evaluating patients’ perceptions on conflicts with decision-making and choosing treatment options (score ranges from 0 to 100; higher scores present a higher decision conflict). In order to assess illness coherence, the five item Illness Perception Scale was used (IPQ-R [43]). The total score ranges from 5 to 25 whereby higher scores present better illness coherence. Additionally the five single items are presented.

In addition, information on healthcare system utilization such as receiving therapies (e.g., operations, radiations therapy, hormone therapy, antibody therapy, targeted therapy, immune therapy, alternative therapies, psychotherapy, or other therapies) and health care services consultations (e.g., counseling regarding work-incapacity and pension, rehabilitation, aids, care counselling, counselling for improvement of living environment, financial advice, psychological support, addiction counseling, or other support) was retrieved [44].

The questionnaire further addressed sociodemographic characteristics (age, gender, family status, care level, and migration background), educational and professional background, social support (OSSS-3 [45], as well as the patients’ perceived social status (MacArthur Scale [46]. The questionnaire was surveyed by face-to-face interviews following patient recruitment in hospital. For follow-up interviews after three, six and twelve months, participants having been offered the choice between a face-to-face, a telephone or a handwritten postal interview. A detailed description of the study design and methods used was previously published [24].

### Statistical analysis

The Intervention and control groups were comparatively characterized with respect to the predefined measures. Thereby, differences between baseline characteristics and outcomes were analyzed using the Chi-square test, Independent t-test, or Mann–Whitney U test, as appropriate. The absolute standardized mean difference (ASMD) was calculated as a standardized effect size to check the balance of the baseline characteristics, whereby ASMD < 0.1 was considered indicating an adequate balance between groups [47]. The level of significance was set to 0.05. All statistical tests were performed using Stata IC15 (StataCorp, 2017, College Station, TX, USA).

## Results

A total of 362 patients were enrolled in the study, with 150 patients belonging to the intervention group and 212 to the control group. Screening data for the intervention group was not available due to data protection in relation of the small number of patients insured in the defined health insurances. For the control group 616 patients were requested. Four patients were excluded from the study; three due to non-compliance and a fourth one declined to participate in the study after enrolment. Differences in baseline characteristics between both groups are shown in Table 1. The participants in the intervention and control groups were unevenly distributed across the study sites. On average, patients in the intervention group were four years older compared to the control group members (66 vs. 62 years, ASMD = 0.0309, *p* = 0.004). Further differences were evident with respect to the diagnoses, such as diagnosis of acute leukemia (23.6% for the control group vs. 12.7% for the intervention group) and lung cancer (13.7% for the control group vs. 22.0% for the intervention group). The proportion of participants with a relative low level of education was 4.7% in the control group, while it was 15.2% in the intervention group (ASMD 0.323, *p* = 0.003). Patients in the control group rated their social status as generally higher than patients in the intervention group (6.4/10 vs. 6.1/10 points, ASMD = 0.223, *p* = 0.055).

**Table 1:** Patients baseline characteristics

**Baseline characteristics** n (%)	**Total** (*n* = 362)	**Intervention** (*n* = 150)	**Control** (*n* = 212)	***p*-value**	**ASMD**
**Study site**				0.002	0.299
Study site 1	119 (32.9%)	43 (28.7%)	76 (35.8%)		
Study site 2	98 (27.1%)	31 (20.7%)	67 (31.6%)		
Study site 3	145 (40.1%)	76 (50.7%)	69 (32.5%)		
**Age** (years)					
Mean (SD) [Min, Max]	63 (13) [19, 85]	66 (13) [24, 85]	62 (13) [19, 85]	0.004	0.309
**Sex**				0.414	0.087
Male	219 (60.5%)	87 (58.0%)	132 (62.3%)		
Female	143 (39.5%)	63 (42.0%)	80 (37.7%)		
**Family status**	*n* = 333	*n* = 124	*n* = 209	0.171	0.186
Married	226 (67.9%)	89 (71.8%)	137 (65.6%)		
Single	44 (13.2%)	18 (14.5%)	26 (12.4%)		
Divorced/Widowed	63 (18.9%)	17 (13.7%)	46 (22.0%)		
**Care level**	*n* = 329	*n* = 123	*n* = 206	0.675	0.022
None	266 (80.9%)	98 (79.7%)	168 (81.6%)		
Yes	63 (19.1%)	25 (20.3%)	38 (18.4%)		
Care level 1	7 (11.1%)	4 (17%)	3 (8%)		
Care level 2	26 (41.3%)	11 (46%)	15 (38%)		
Care level 3	21 (33.3%)	6 (25%)	15 (38%)		
Care level 4	9 (14.3%)	3 (13%)	6 (15%)		
**Time since diagnosis** (months)					
Median (P25, P75)	6 (2, 22)	5 (1, 26)	7 (2, 19)	0.416	0.114
< 12 months	227 (62.7%)	89 (59.3%)	138 (65.1%)		
12 – 60 months	108 (29.8%)	46 (30.7%)	62 (29.2%)		
> 60 months	27 (7.5%)	15 (10.0%)	12 (5.7%)		
**Diagnosis**				0.013	0.247
Acute leukemia	69 (19.1%)	19 (12.7%)	50 (23.6%)		
Aggressive lymphoma	58 (16.0%)	20 (13.3%)	38 (17.9%)		
Malignant neoplasm of bronchus and lung	62 (17.1%)	33 (22.0%)	29 (13.7%)		
Metastasized colorectal cancer	78 (21.6%)	34 (22.7%)	44 (20.8%)		
Malignant neoplasm of pancreas	32 (8.8%)	17 (11.3%)	15 (7.1%)		
Multiple myeloma and malignant plasma cell neoplasms	24 (6.6%)	6 (4.0%)	18 (8.5%)		
Metastasized malignant neoplasm of breast	9 (2.5%)	4 (2.7%)	5 (2.4%)		
Others	30 (8.3%)	17 (11.3%)	13 (6.1%)		
**Duration of living in Germany** (years)	*n* = 332	*n* = 125	*n* = 207	0.762	0.050
Since birth	287 (86.5%)	107 (85.6%)	180 (87.0%)		
< 5 years	4 (1.2%)	1 (0.8%)	3 (1.4%)		
≥ 5 years	41 (12.3%)	17 (13.6%)	24 (11.6%)		
**Education**	*n* = 336	*n* = 125	*n* = 211	0.003	0.323
Low	29 (8.6%)	19 (15.2%)	10 (4.7%)		
Medium	101 (30.1%)	38 (30.4%)	63 (29.9%)		
High	206 (61.3%)	68 (54.4%)	138 (65.4%)		
**Financial Situation**	*n* = 331	*n* = 123	*n* = 208	0.817	0.022
Very good	28 (8.5%)	9 (7.3%)	19 (9.1%)		
Good	173 (52.3%)	65 (52.8%)	108 (51.9%)		
Moderate	91 (27.5%)	37 (30.1%)	54 (26.0%)		
Difficult	28 (8.5%)	9 (7.3%)	19 (9.1%)		
Very difficult	11 (3.3%)	3 (2.4%)	8 (3.8%)		
**Subjective Social Status (MacArthur Scale)**	*n* = 314	*n* = 122	*n* = 201		
Mean (SD)	6.3 (1.5)	6.1 (1.4)	6.4 (1.5)	0.055	0.223
**Social support (OSSS-3)**	*n* = 332	*n* = 124	*n* = 208		
Mean (SD)	11.0 (2.1)	10.8 (2.1)	11.1 (2.1)	0.300	0.118
Poor (3 – 8)	35 (10.5%)	15 (12.1%)	20 (9.6%)		
Moderate (9 – 11)	158 (47.6%)	60 (48.4%)	98 (47.1%)		
Strong (12 – 14)	139 (41.9%)	49 (39.5%)	90 (43.3%)		

*ASMD* Absolute standardized mean difference, *Ref*. Reference category was omitted, care level 1 = minor impairment of independence, Care level 4 = most severe impairment of independence

**Fig.1:**
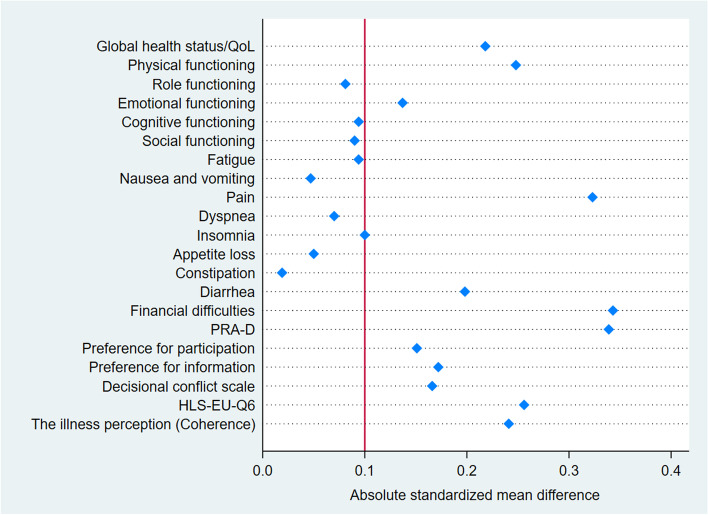
Absolute standardized mean difference between intervention and control groups. Absolute values of standardized differences < 0.1 indicated sufficient balance

Overall, patients belonging to the control group reported better global quality of life and physical functioning as in comparison to those in the intervention group (Table 2). The mean QoL was 52.6 (SD = 21.7) in the control group and 47.8 (SD = 22.0) in the intervention group (ASMD = 0.218, *p* = 0.044). Furthermore, patients in the intervention group reported worse outcomes in the symptom scale for pain and fatigue than the control group. Participants in the control group reported financial difficulties more often compared to patients in the intervention group, with a mean difference of 10 points between the two groups (ASMD = 0.343, *p* = 0.002). The proportions of the quality of life (EORTC QLQ-C30), functioning scales and symptom burden at baseline are summarized in Table 2 and illustrated in Fig. 1.

**Table 2:** EORTC QLQ-C30 scores at baseline

EORTIC QLQ-C30	Total		Intervention		Control		*p*-value	ASMD
	**n**	**Mean (SD)**	**n**	**Mean (SD)**	**n**	**Mean (SD)**		
**Global health status/QoL**	355	50.6 (21.9)	147	47.8 (22.0)	208	52.6 (21.7)	0.044	0.218
**Funktional**								
Physical functioning	358	57.6 (25.4)	149	54.0 (24.3)	209	60.2 (25.9)	0.022	0.248
Role functioning	359	40.6 (33.8)	147	39.0 (33.8)	212	41.7 (33.7)	0.450	0.081
Emotional functioning	357	57.2 (27.1)	149	55.0 (26.3)	208	58.7 (27.6)	0.205	0.137
Cognitive functioning	358	73.9 (29.1)	149	75.5 (27.2)	209	72.8 (30.4)	0.388	0.094
Social functioning	357	49.7 (35.1)	149	51.6 (34.4)	208	48.4 (35.6)	0.401	0.090
**Symptom Scale**								
Fatigue	359	57.7 (28.9)	148	59.3 (28.2)	211	56.6 (29.5)	0.382	0.094
Nausea and vomiting	360	17.4 (26.7)	148	16.7 (27.1)	212	17.9 (26.5)	0.661	0.047
Pain	361	37.3 (36.1)	149	44.1 (36.8)	212	32.5 (35.0)	0.003	0.323
Dyspnoea	360	39.4 (38.3)	149	40.9 (39.2)	211	38.2 (37.7)	0.509	0.070
Insomnia	360	43.9 (38.9)	149	41.6 (39.1)	211	45.5 (38.8)	0.352	0.100
Appetite loss	359	39.6 (39.1)	147	40.8 (40.1)	212	38.8 (38.5)	0.638	0.050
Constipation	360	21.9 (33.8)	149	21.5 (32.9)	211	22.1 (34.5)	0.860	0.019
Diarrhoea	361	21.9 (32.7)	149	18.1 (30.9)	212	24.5 (33.7)	0.066	0.198
**Item**								
Financial difficulties	357	17.9 (30.4)	149	12.1 (23.6)	208	22.1 (33.9)	0.002	0.343

*ASMD* Absolute standardized mean difference

Results regarding the secondary outcomes at baseline are provided in Table 3 whereby a ASMD < 0.1 indicates balanced distribution of characteristics between intervention and control group. A significant difference was observed between both groups with respect to scores of doctor-patient communication (PRA-D). The mean of PRA-D was higher in the control group compared to the intervention group (30.6 vs. 28.4, ASMD = 0.339, *p* = 0.003). Moreover, the median of Decisional Conflict Scale (DCS) showed to be 20 points (IQR = 5 to 42) for those who were exposed to the intervention and 15 (IQR = 0 to 35) for participants of the control group, indicating more decisional conflicts in the intervention group. Inadequate health literacy was reported by 22.8% of patients in the intervention group and 11.0% in the control group. In line with these findings, 31.6% of patients in the intervention group and 40.2% of those belonging to the control group showed sufficient health literacy (ASMD = 0.256, *p* = 0.064). Furthermore, the control group scored higher on the Coherence of Disease Subscale (mean score 16.6 vs. 15.6, ASMD = 0.241, *p* = 0.038). No substantial group differences were found for the API-DM scale – neither regarding patients’ information preferences (mean score 96.5 compared to 95.4, ASMD = 0.172, *p* = 0.118) nor for participation preferences (mean score 53.7 compared to 51.5, ASMD = 0.151, *p* = 0.185).

**Table 3:** Secondary outcomes at baseline

Secondary outcomes	Total	Intervention	Control	*p*-value	ASMD
**Doctor-Patient Communication (PRA-D)**	*n* = 335	*n* = 124	*n* = 211		
Mean (SD)	29.8 (6.5)	28.4 (7.0)	30.6 (6.0)	0.003	0.339
**Autonomy Preference Index scores (API-DM)**					
**Preference for participation**	*n* = 336	*n* = 127	*n* = 209		
Mean (SD)	52.8 (14.9)	51.5 (14.1)	53.7 (15.3)	0.185	0.151
**Preference for information**	*n* = 337	*n* = 126	*n* = 207		
Mean (SD)	96.1 (6.5)	95.4 (7.3)	96.5 (6.0)	0.118	0.172
**Decisional Conflict Scale (DCS)**	*n* = 335	*n* = 124	*n* = 211		
Median (IQR)	15 (0, 40)	20 (5, 42)	15 (0, 35)	0.046	0.166
**Health literacy (HLS-EU-Q6)**	*n* = 243	*n* = 79	*n* = 164		
Mean (SD)	2.8 (0.7)	2.6 (0.6)	2.8 (0.7)	0.064	0.256
(1-2) = inadequate HL	36 (14.8%)	18 (22.8%)	18 (11.0%)		
(2–3) = problematic HL	116 (47.7%)	36 (45.6%)	80 (48.8%)		
(3-4) = sufficient HL	91 (37.5%)	25 (31.6%)	66 (40.2%)		
**The Illness Perception – Coherence (IPQ-R)**	*n* = 332	*n* = 123	*n* = 209		
Mean (SD)	16.2 (4.3)	15.6 (4.0)	16.6 (4.5)	0.038	0.241
**I cannot explain the symptoms of my illness**	*n* = 331	*n* = 123	*n* = 208	0.047	0.085
Not at all true	69 (20.9%)	17 (13.8%)	52 (25.0%)		
Not true	95 (28.7%)	40 (32.5%)	55 (26.4%)		
Neither true nor not	42 (12.7%)	19 (15.5%)	23 (11.1%)		
True	76 (23.0%)	33 (26.8%)	43 (20.7%)		
Completely true	49 (14.8%)	14 (11.4%)	35 (16.8%)		

*ASMD* Absolute standardized mean difference, *HL* Health literacy

Patients’ utilization of therapies and health care services during the last three months before study enrolment differed across patient groups (see Table 4). In the control group, higher usage rates of antibody therapy, targeted therapy, and chemotherapy were reported compared to the intervention group (each ASMD > 0.2). Similarly, patients in the control group were found to use health care support – such as disability and pension counseling, rehabilitation, therapeutic or medical aids, and psychological support – more frequently than participants belonging to the intervention group.

**Table 4:** Therapy and support in the last 3 months before enrollment

	**Total**	**Intervention**	**Control**	***p*-value**	**ASMD**
**Therapy**^a^					
Operation	91 (29.1%)	33 (28.7%)	58 (29.3%)	0.911	0.013
Radiation therapy	43 (13.9%)	19 (16.7%)	24 (12.3%)	0.285	0.124
Chemotherapy	248 (76.1%)	84 (70.0%)	164 (79.6%)	0.050	0.222
Hormone therapy	11 (3.6%)	4 (3.5%)	7 (3.6%)	0.968	0.005
Antibody therapy	64 (20.6%)	15 (12.8%)	49 (25.3%)	0.009	0.320
Targeted therapy	30 (9.8%)	5 (4.4%)	25 (13.1%)	0.014	0.311
Immune therapy	33 (10.7%)	11 (9.6%)	22 (11.3%)	0.636	0.056
Alternative therapies	1 (0.3%)	0 (0.0%)	1 (0.5%)	0.446	0.101
Psychotherapy	26 (8.4%)	9 (7.9%)	17 (8.7%)	0.801	0.030
Physiotherapy	57 (18.3%)	19 (16.5%)	38 (19.4%)	0.528	0.074
Other therapies	12 (4.2%)	3 (3.0%)	9 (4.8%)	0.480	0.090
**Support**^a^					
Counseling on work-incapacity and pension	32 (9.9%)	5 (4.2%)	27 (13.2%)	0.010	0.320
Rehabilitation	21 (6.5%)	4 (3.4%)	17 (8.3%)	0.088	0.208
Aids, including care aids	55 (17.0%)	14 (11.8%)	41 (20.0%)	0.057	0.226
Care counseling	52 (16.1%)	19 (16.0%)	33 (16.1%)	0.975	0.004
Counseling for the improvement of the individual living environment	15 (4.7%)	5 (4.2%)	10 (4.9%)	0.785	0.032
Financial advice	11 (3.4%)	3 (2.5%)	8 (3.9%)	0.521	0.076
Psychological support	45 (14.1%)	10 (8.5%)	35 (17.2%)	0.031	0.261
Addiction counseling	1 (0.3%)	0 (0.0%)	1 (0.5%)	0.447	0.099
Other support	11 (3.7%)	5 (4.7%)	6 (3.1%)	0.501	0.079

*ASMD* Absolute standardized mean difference, ^a^Data of therapy and support including missings

## Discussion

The aim of the analysis of the OSCAR-study baseline characteristics was to identify and discuss possible systematic differences between the two study groups. Our comparative analysis revealed that the recruited patients were not evenly distributed to the intervention and control groups, respectively. More specifically, the study site location, age, diagnosis, QoL, doctor-patient communication, illness perception, and the educational background varied considerably between the two groups. Thereby, the results indicate a higher social and health-related vulnerability of the patients in the intervention group compared to those in the control group. Such a comparative analysis is valuable to assess the comparability of results between the intervention and control groups and to develop appropriate statistical analysis procedures for existing group differences.

### Measurement of patient-reported outcomes in the OSCAR-study

At baseline, patients belonging to the control group reported a better global health status and quality of life, as well as lower symptom burdens as in comparison to the intervention group. As to be expected, QoL values measured in OSCAR were below the average found in a representative sample of the German general population (71.5 points, [48]) and European reference values (75.7 points) [49]. However, the values are lower for both groups compared to those of other studies: For instance, in a study on patients undergoing immunotherapy or chemotherapy treatments the average QoL score was 62.6 points [50]. Similarly, a German study of oncological patients, who were interviewed six months after a rehabilitation stay achieved an average score of 69.3 points [51]. QoL values measured in the OSCAR Study are comparable to the quality of life of palliative oncology patients in the last year of life [52]. The comparatively low scores in the intervention as well as control groups emphasize the relatively high burden of disease among the participating patients. Furthermore, social support – measured by means of the OSSS-3 scale – showed to be similar to mean values in the general German population [45]. The transformed values of our version of the PRA-D, which was adapted to assess doctor-patient communication, were higher than (regarding the control group) or comparable with (regarding the intervention group) reference values of the PRA-D [40]. In the light of the publication by Brenk-Franz et al., the baseline doctor-patient-communication in OSCAR can be interpreted as mostly satisfactory for both groups.

With respect to potential decision conflicts (DCS-score), results from our intervention and control groups indicate a low potential for decision conflicts about the hypothetical decision between treatment alternatives. The majority of patients felt relatively confident about their potential decision and thus seemed to be aware of the (dis)advantages of one therapy or another. In contrast, a validation of this instrument in a study on 149 patients with prostate cancer screening showed higher DCS-values at baseline [53]. These patients had more potential for internal conflicts relating to decisions about screening plans in the future as compared to the OSCAR-patients.

Results from the Participation and Information Preference Instrument (API-DM) indicated a strong need for information about the course of the disease and treatment as well as different therapy options for both the intervention and the control group. Additionally, both groups showed a similar preference for their involvement in medical decisions. Thereby, patients preferred a shared decision-making process together with their attending physician. For both categories, a French validation study of API for cancer patients found lower results (information preference = 85.3/100; participation preference = 45.6/100) [54]. The reference values for health literacy in the general German population (HLS-EU-6 [55]) largely correspond to those of the OSCAR control group. However, in the intervention group, the proportion of patients, who were classified in the “inadequate” category, was twice as high compared to the control group.

### Understanding the differences in baseline characteristics

#### Benefits of participation

Having identified differences between the control and intervention groups the question arises, how those discrepancies can be explained. Following the observations of previous studies, we have to assume that participation in intervention studies is a challenge and selective in any case [26, 56]. Reasons for (non-)selection can be identified on the patient side as well as on the recruiter side: With respect to the recruitment, older patients with comorbid conditions, for example, were shown to be less likely to be offered participation [27]. On the patient side, Cottin et al. showed that severely affected patients are less likely to participate in trials [57]. Moreover, non-included patients show a significantly higher symptom-related limitation of activity, comorbidity, and lower self-determination, whereby older ones had a lower response rate to treatment and a shorter rate of survival [26, 57]. The OSCAR-study focused on patients with advanced cancers, whose disease burden and thereby affected life situations made a reduced study participation expectable. At the same time, participation in the study promised a concrete benefit for the patients in the intervention, which could lead to recruitment of sicker patients compared to the control group. As Puts et al. and Wright et al. showed, a higher willingness to participate in treatment and trials depend on the individually perceived benefit, probable success rate of treatment, side effects as well as the support offered in making a decision regarding trial entry—in our case, this support was provided by the personal social care nurses [28, 29].

#### The price for the required sample size

Recruiting participants for surveys among vulnerable and weakened patient populations is more resource-intensive, however is limited by the preset project durations and funding situation. In OSCAR, patients with multiple indications – each indicative of an advanced cancer – were eligible for study participation. The inclusion of such a broad range of diagnoses increases the number of potential study participants, while also raising the risk of bias between the intervention and control groups, especially if not all diagnoses are identical in their course of disease, symptom burden and prognosis [58]. Previous studies showed clear differences across tumor types with respect to symptoms and supportive care needs, specifically for respiratory in contrast to hematopoietic/lymphatic disease sites. These entities were not equally distributed between intervention and control group in OSCAR and might explain a bias towards a lower symptom burden in patients of the control group.

#### The role of funding conditions

Furthermore, the recruitment criterion of membership in certain health insurance companies limited access to the intervention group, which did not apply to the control group. Randomization into either of the two groups could not be realized and may thus have been a potential source of selection bias. The reason for this is the special funding framework within which the current study took place – the innovation fund of the German Federal Joint Committee. The existing economic competition between the types of health insurance funds in Germany favors the pursuit of individual projects and initiatives in order to distinguish themselves from one another and to be able to offer their own and potential new members particularly innovative services. The health insurance funds in Germany are characterized by different social and morbidity structures due to the historical background of their emergence [59, 60]. Basically, a distinction can be made between five types of health insurance funds: employees' health insurance fund, company health insurance funds, guild health insurance funds, miners' health insurance, local health insurance funds. Although the former allocation law has been replaced, according to which most statutory health insurance members were allocated to a specific health insurance fund depending on the characteristics of their workplace, the differences in the patient clientele persist [59].

Although patients in the intervention group were insured in 37 different health insurance funds, all of these are part of the same type of insurance (company health insurance funds). Hoffman & Icks pointed to differences in the distribution of person characteristics such as age, gender, weight, education level, as well as of the health status, smoking behavior, and specific diseases that varied by insurance type [59]. Those findings are meaningful in contextualizing our baseline findings. Their observations regarding higher proportion of women and lower educated in that type of insurance could be confirmed. Additionally, higher consumption rates in smokers were reported before, which we did not assess. The differences in the social composition and health behavior in the intervention group are well explored health predictors and may therefore be well related to higher symptom burdens in the intervention groups [61].

### Study site effects

In addition, there were regional differences in the proportion of health insurance memberships among the recruited study participants. While the control group was enrolled equally at all three study sites, half of the patients in the intervention group were recruited at the third study site. This hospital had a palliative focus, which may be reflected in a higher percentage of patients being severely affected and particularly vulnerable. Although symptom burden and supportive care needs are often already high at the time of diagnosis, studies could demonstrate an increase of symptoms and decline of functioning with disease progression and increasing number of therapies of cancer patients [62]*.*

### Differences by chance

In relation to a cancer diagnosis, stratified randomization should lead to an evenly balanced distribution of known and unknown characteristics in the intervention and control groups. However, even in a randomized study design significant differences between study groups can occur, as in the study by Wagner et al., for instance, which examined the influence of nursing navigators on patients’ QoL [19]. This was the case for the distribution of educational qualifications within the control and intervention group in the context of cluster randomization. The authors also noted that cluster randomization prevented the random distribution of diagnoses within the groups [19]. More generally, Deaton and Cartwright pointed out that randomization procedures in medical intervention studies primarily determine whether patients are assigned to the intervention or control group but do not automatically lead to a random distribution of outcomes [63]. Irrespective of whether randomization is performed or not, it seems advisable to control for the baseline distribution of patients characteristics [30–32]. Despite non-randomized trials are inherently more prone to bias this does not necessarily result in different intervention effects due to adjustment for confounders or baseline imbalances [33, 34, 64]. A Cochrane review of methodological papers that comparatively analyzed healthcare outcomes from randomized studies and observational studies revealed only limited evidence for significant differences in the results of both study types [64].

### Limitations and approaches to statistical address group differences

As shown above, our results show a higher general quality of life and lower symptom burden in the control group as in comparison to the intervention group. Such a phenomenon can bias study outcomes, and it seems likely that these differences will result in a substantial bias in the intervention effect [65]. However, the study has strengths in areas that are important for the generalizability of the results. The participating hospitals represent the diversity of the oncology care landscape, as university hospitals and (supra)regional hospitals are involved. Although rural areas appear to be underrepresented.

Adjustments for unbalanced sample distributions can be accomplished by several common statistical methods, including matched sampling, stratification, and multiple regression models. However, restrictions on the number of covariates to be considered often pose a challenge [65]. Propensity Score Methods are a set of methods for reducing such bias and has become popular for observational studies. The four common methods used in the propensity score are matching, stratification, covariate adjustment, and Inverse Probability of Treatment Weighting (IPTW). Regarding the limited sample size in the OSCAR study, the IPTW method using all available data will be considered in future analyses. The use of this method is advantageous because it allows the inclusion of all participants and reduces bias more effectively compared to other adjustment methods such as stratification and covariate adjustment [66]. A difficulty that can arise is the presence of large weights assigned to groups which could be assigned to those observations [67]. To address this problem, the use of stabilized, trimmed or truncated weights will be considered for further analyses of the OSCAR data [67, 68]. In addition, sensitivity analyses will stratify the results by diagnosis and study site, enabling the identification of selection effects during the recruitment phase. Finally, the sample will be repeatedly characterized at each follow-up point to identify potential changes in the composition of the study groups.

## Conclusion

Intervention studies are commonly confronted with recruitment requirements and difficulties that favor selection effects, especially when severely ill patients with a poor prognosis are to be involved. Comparative analyses of central context and patient characteristics at baseline facilitate the identification of systematic differences between study groups and allow the reasons for such differences to be uncovered and discussed. Unobserved group differences may preclude a valid evaluation and correct judgement of the effectiveness of the intervention being tested. Against this background, a monitoring procedure accompanying the recruitment phase is recommended, by means of which the distribution of essential characteristics such as the study site and various participant characteristics (age, gender, or health status) are systematically verified to detect potential selection effects. Such a procedure may optimize the recruitment process in intervention studies, since appropriate measures can eventually be taken to adequately address identified selection effects as well as to reduce their occurrence in the first place. Moreover, in order to establish a valid comparability between different study groups, any differences at baseline need to be accounted for in the statistical analyses by means of appropriate methodological measures.

## Supplementary Information

**Additional file 1.** Included ICD-10- and OPS-codes.

## Data Availability

The datasets generated and analyzed during the current study are not publicly available since the storage of the data in a public register is not covered by the patients’ declaration of consent. An anonymous data set are however available upon reasonable request. The corresponding author [DS] will provide data, analytical codes and material.

## Abbreviations

API-DM: German modified version of the Autonomy Preference Index
ASMD: Absolute standardized mean difference
DCS: Decision Conflict Scale
DRKS: German Clinical Trials Register
EORTC: European Organization for Research and Treatment of Cancer
HLS-EU-Q6: European Health Literacy Survey
ICD: International Statistical Classification of Diseases and Related Health Problems
IPQ: Illness Perception Questionnaire
IPTW: Inverse probability of treatment weighting
IQR: Interquartile range
OECD: Organization for Economic Co-operation and Development
OPS: Operation and Procedure Codes (German adaption of the International Classification of Procedures in Medicine (ICPM))
OSCAR: Oncological Social Care Project
OSSS-3: Oslo Social Support Scale
PRA-D: Patient Reaction Assessment
PRO: Patient-reported outcome
QoL: Quality of life
SCN: Social Care Nurse
SD: Standard deviation
