# Supplementary material for: Identifying and handling unbalanced baseline characteristics in a non-randomized, controlled, multicenter social care nurse intervention study for patients in advanced stages of cancer
Source: BMC Cancer. 2022 May 18;22:560. doi: 10.1186/s12885-022-09646-6 (PMC9118792; doi:10.1186/s12885-022-09646-6)
Supplement: Supplementary file 1 — Additional file 1. Included ICD-10- and OPS-codes. [file 12885_2022_9646_MOESM1_ESM.docx]

**Identifying and handling unbalanced baseline characteristics in a non-randomized, controlled, multicenter social care nurse intervention study for patients in advanced stages of cancer**

Johann Frick^1^, Pimrapat Gebert^1,2,3^, Ulrike Grittner^2,3^, Anne Letsch^4,5^, Daniel Schindel*^1^, and Liane Schenk*^1^

*these authors contributed equally

^1^ Charité – Universitätsmedizin Berlin, corporate member of Freie Universität Berlin, Humboldt-Universität zu Berlin, and Berlin Institute of Health, Institute of Medical Sociology and Rehabilitation Science, Charitéplatz 1, 10117 Berlin, Germany

^2^ Charité – Universitätsmedizin Berlin, corporate member of Freie Universität Berlin, Humboldt-Universität zu Berlin, and Berlin Institute of Health, Institute of Biometry and Clinical Epidemiology, Charitéplatz 1, 10117 Berlin, Germany

^3^ Berlin Institute of Health (BIH), Anna-Louisa-Karsch-Str. 2, 10178 Berlin, Germany

^4^ University Hospital Schleswig-Holstein, Department of Medicine II, Hematology and Oncology, Arnold-Heller-Straße 3, 24105 Kiel, Germany

^5^ Charité – Universitätsmedizin Berlin, corporate member of Freie Universität Berlin, Humboldt-Universität zu Berlin, and Berlin Institute of Health, Charité Comprehensive Cancer Center, Charitéplatz 1, 10117 Berlin, Germany

**Additional file 1: Included ICD-10- and OPS-codes**

| **Summary of ICD 10-codes with 5 digits** | |
| --- | --- |
| Metastasized malignant neoplasms of lip, oral cavity, and pharynx | C00 – C14 and C77 – C79 |
| Malignant neoplasm of esophagus | C15 |
| Malignant neoplasm of stomach | C16 |
| Metastatic colorectal cancer / colon carcinoma | C18 – C20 and C77 – C79 |
| Malignant neoplasm of pancreas | C25 |
| Malignant neoplasm of bronchus and lung | C34 |
| Metastasized melanoma and other malignant neoplasms of skin | C43 – C44 and C77 – C79 |
| Metastasized malignant neoplasm of breast | C50 and C77 – C79 |
| Metastasized malignant neoplasm of ovary | C56 and C77 – C79 |
| Metastasized malignant neoplasm of cervix uteri | C53 and C77 – C79 |
| Metastasized malignant neoplasm of corpus uteri | C54 and C77 – C79 |
| Metastasized malignant neoplasm of prostate | C61 and C77 – C79 |
| Metastasized malignant neoplasm of thyroid gland | C73 and C77 – C79 |
| Lymphoma | C82 – C86 |
| Multiple myeloma and malignant plasma cell neoplasms | C90 |
| Leukemia | C91 – C92 |

ICD-10-codes: International Statistical Classification of Diseases and Related Health Problems (Version 10)

| **Summary of OPS-codes** | |
| --- | --- |
| Surgical operation on the digestive tract | 5-42 – 5-54 |
| Surgical operation on the lymphatic tissues | 5-402 – 5-404  5-406 |
| Radiotherapy, nuclear medicine therapy and pain management | 8-52  8-53  8-91 |
| Multimodal pain treatment, cytotoxic chemotherapy, complex treatment | 8-541 – 8-544  8-546  8-918  8-982  8-98e |

OPS-codes: Operation and Procedure Codes (German adaption of the International Classification of Procedures in Medicine by the WHO (ICPM))
